# Gallic acid rescues uranyl acetate induced-hepatic dysfunction in rats by its antioxidant and cytoprotective potentials

**DOI:** 10.1186/s12906-023-04250-y

**Published:** 2023-11-22

**Authors:** Ibtisam M. H. Elmileegy, Hanan S. A. Waly, Alshaimaa A. I. Alghriany, Nasser S. Abou Khalil, Sara M. M. Mahmoud, Eman A. Negm

**Affiliations:** 1 Department of Medical Physiology, Faculty of Medicine, Assiut University, Assiut, 71526 Egypt; 2 Laboratory of Physiology, Department of Zoology and Entomology, Faculty of Science, Assiut University, Assiut, Egypt; 3 Department of Zoology and Entomology, Faculty of Science, Assiut University, Assiut, Egypt; 4 Department of Basic Medical Sciences, Faculty of Physical Therapy, Merit University, Sohag, Egypt; 5 Department of Physiology, Faculty of Veterinary Medicine, Assiut University, Assiut, 71526 Egypt

**Keywords:** Uranyl acetate, Gallic acid, Liver, Physiology, Nrf2, Apoptosis

## Abstract

**Background:**

The liver was identified as a primary target organ for the chemo-radiological effects of uranyl acetate (UA). Although the anti-oxidant and anti-apoptotic properties of gallic acid (GA) make it a promising phytochemical to resist its hazards, there is no available data in this area of research.

**Methods:**

To address this issue, eighteen rats were randomly and equally divided into three groups. One group was received carboxymethyl cellulose (vehicle of GA) and kept as a control. The UA group was injected intraperitoneally with UA at a single dose of 5 mg/kg body weight. The third group (GA + UA group) was treated with GA orally at a dose of 100 mg/kg body weight for 14 days before UA exposure. UA was injected on the 15th day of the experiment in either the UA group or the GA + UA group. The biochemical, histological, and immunohistochemical findings in the GA + UA group were compared to both control and UA groups.

**Results:**

The results showed that UA exposure led to a range of adverse effects. These included elevated plasma levels of aspartate aminotransferase, lactate dehydrogenase, total protein, globulin, glucose, total cholesterol, triglycerides, low-density lipoprotein cholesterol, and very-low-density lipoprotein and decreased plasma levels of high-density lipoprotein cholesterol. The exposure also disrupted the redox balance, evident through decreased plasma total antioxidant capacity and hepatic nitric oxide, superoxide dismutase, reduced glutathione, glutathione-S-transferase, glutathione reductase, and glutathione peroxidase and increased hepatic oxidized glutathione and malondialdehyde. Plasma levels of albumin and alanine aminotransferase did not significantly change in all groups. Histopathological analysis revealed damage to liver tissue, characterized by deteriorations in tissue structure, excessive collagen accumulation, and depletion of glycogen. Furthermore, UA exposure up-regulated the immuno-expression of cleaved caspase-3 and down-regulated the immuno-expression of nuclear factor-erythroid-2-related factor 2 in hepatic tissues, indicating an induction of apoptosis and oxidative stress response. However, the pre-treatment with GA proved to be effective in mitigating these negative effects induced by UA exposure, except for the disturbances in the lipid profile.

**Conclusions:**

The study suggests that GA has the potential to act as a protective agent against the adverse effects of UA exposure on the liver. Its ability to restore redox balance and inhibit apoptosis makes it a promising candidate for countering the harmful effects of chemo-radiological agents such as UA.

## Background

The enduring presence of depleted uranium (DU) in bio-ecological systems, its diverse entry routes, magnification through the food chain, and the compounded effects of both metallic and radiation toxicities [1, 2] establish it as a prominent environmental pollutant. Among vulnerable organs to DU-related issues, the liver stands out due to its role as a center for xenobiotic accumulation and metabolism [3]. The intoxication with uranyl acetate (UA) led to a breakdown of the protective antioxidant shield in the liver, primarily driven by reduced nuclear translocation of nuclear factor-erythroid-2-related factor 2 (Nrf2). UA-induced molecular changes resulting in apoptosis within the liver involved the activation of caspase-3, elevation of Bcl-2/Bax ratio, release of cytochrome c from mitochondria, and decrease in ATP levels, all collectively promoting cellular death [3–5]. The attack by free radicals and initiation of the apoptotic pathway lead to degenerative and necrotic modifications in hepatocytes, subsequently releasing liver metabolic enzymes into the bloodstream [4]. Thus, employing bioactive compounds with antioxidant and cytoprotective properties could potentially counteract UA-induced hepatotoxicity. Although sequestering agents have been widely used to counteract UA radiotoxicity, they often yield unsatisfactory outcomes due to their nonspecific affinity, limited efficacy, insufficient clinical trials, and potential to induce acid-base imbalance and renal toxicity [1, 6, 7].

These challenges are driving a new wave of research focused on natural biological approaches to mitigate chemo-radiological risks posed by UA. Our laboratory demonstrated the effectiveness of thymoquinone and N-acetylcysteine against UA-induced testicular damage in rats, primarily through their anti-apoptotic and cytoprotective mechanisms rather than their antioxidant properties [8]. Substantial evidence from animal models and cell cultures supports the protective potential of gallic acid (GA) on irradiated livers. Supplementation of mice exposed to gamma rays with GA prevented the depletion of antioxidant defenses and excessive lipid peroxidation in the liver [9]. However, the impact of GA on hepatic metabolic enzyme activity remains unstudied. Ferk et al. [10] reported that GA intervention alleviated gamma radiation-induced genotoxic damage and preneoplastic foci in rats. They attributed these effects to the antioxidant potency of GA, believed to stem from redox-related transcription regulator up-regulation, without solid molecular evidence. Other studies using a mouse model of dimethylnitrosamine-induced hepatotoxicity revealed that GA increased Nf2 transcript levels, which subsequently bound to DNA sequences to activate redox stabilizers’ expression [11]. Nonetheless, whether GA can mitigate UA-induced hepatic dysfunction remains uncertain. Hence, this study aims to address this gap by evaluating potential changes in plasma metabolic enzymes, liver redox homeostasis, histological features, as well as caspase-3 and Nrf2 immuno-expression in Wistar rats.

## Methods

### Drugs and chemicals

UA dihydrate (purity ≥ 98% and molecular weight 424.15 g/mol) was purchased from Sigma-Aldrich Company (St. Louis, MO, USA). GA (purity ≥ 99%) was obtained from Sd Fine Chem. Limited Company, India. Carboxymethyl cellulose (CMC) 98% CAS: 9005-64-5 was obtained from Alpha Global Search Company, New York, USA.

### Experimental animals

A total of 18 adult male Wistar rats were used for this study. The rats were purchased from the Egyptian Company for Production of Vaccines, Sera, and Drugs, Egypt, and housed under natural light/dark cycles, at a temperature of 20–25 °C, and relative humidity of 55.0 ± 5.0%. They were provided with commercial pelleted feed and water *ad libitum*.

### Experimental design

After a one-week acclimatization period, the rats were randomly divided into three groups, each consisting of six animals. The control group received carboxymethyl cellulose (CMC); the vehicle of GA. The second group (UA group) received an intraperitoneal injection of UA at a single dose of 5 mg/kg body weight [4]. The third group (GA + UA group) was orally administered GA using a stomach tube at a dose of 100 mg/kg body weight [12] dissolved in 1% CMC for 14 days prior to UA exposure. UA was injected on the 15th day of the experiment in either the UA group or the GA + UA group.

### Collection and preparation of samples

**Fig. 1:**
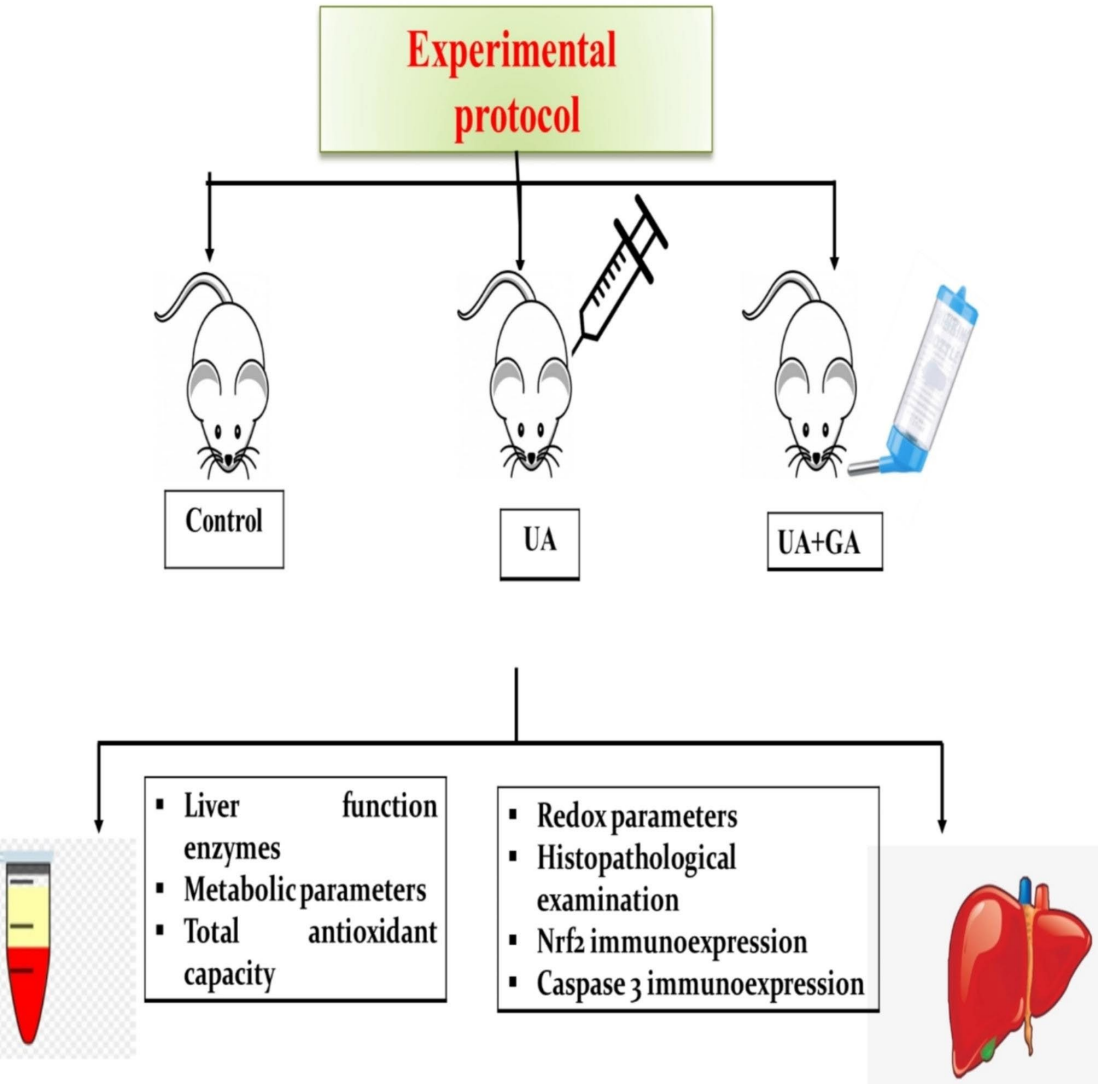
Graphical representation of the experimental procedure. UA: uranyl acetate; GA: gallic acid; Nrf2: nuclear factor-erythroid-2-related factor 2

At the end of the experimental period, blood samples were collected from the retro-orbital sinus after overnight fasting. Plasma was separated by centrifugation at 3000 rpm for 10 min and stored at -20 °C for subsequent biochemical analyses. Rats were euthanized by cervical dislocation under anesthesia induced by intraperitoneal injection of sodium thiopental. Half of the liver was promptly excised, homogenized in 1 ml of 0.1 M phosphate buffer (pH 7.4), and centrifuged at 10,000 rpm for 15 min using IKA Yellow line DI homogenizer (18 Disperser, Germany) to give 10% w/v homogenate. The homogenates were centrifuged at 10.000 rpm for 15 min, and the resulting supernatants were frozen at -20 °C for measurement of oxidant/antioxidant parameters. The other half of liver was fixed in 10% neutral buffered formalin for histopathological evaluation. The experimental procedure was represented in Fig. 1.

### Biochemical measurements

Plasma alanine aminotransferase (ALT) (Catalog number: 264001), aspartate aminotransferase (AST) (Catalog number: 260001), albumin level (Catalog number: 211001), total protein (Catalog number: 310001), glucose (Catalog number: 250001), total cholesterol (TC) (Catalog number: 230002), triglyceride (TG) (Catalog number: 314002), and high-density lipoprotein cholesterol (HDL-C) (Catalog number: 266001) were assessed according to the manufacturer’s instructions using commercial kits provided by Egyptian Company for Biotechnology Company, Egypt. Plasma low-density lipoprotein cholesterol (LDL-C) was determined using the Friedewald formula: LDL-C = TC – HDL-C – [TG/5] [13]. Plasma very-low-density lipoprotein (VLDL) was calculated according to [14].

Total globulins were calculated by subtracting the obtained albumin level from the obtained total proteins level [15]. Plasma lactate dehydrogenase (LDH) activity was measured by a kinetic method using a commercial kit (Catalog number: 2940, Stabino Laboratory Company, Texas, Egypt). The levels of malondialdehyde (MDA) were measured by thiobarbituric acid reaction according to the procedure of Ohkawa et al. [16]. Nitric oxide (NO) was measured as nitrite concentration using the method of Ding et al. [17]. Total antioxidant capacity (TAC) was measured using a calorimetric kit (Catalog number: TA2513, Biodiagnostic, Giza, Egypt). Superoxide dismutase (SOD) activity was determined based on its inhibition of epinephrine autoxidation [18]. Reduced glutathione (GSH) content was estimated using the method of Beutler et al. [19]. Oxidized glutathione (GSSG) levels were measured by the enzymatic recycling method described by Tietze [20]. Glutathione peroxidase (GPx) activity was determined by measuring the decrease in GSH content after incubating the sample in the presence of hydrogen peroxide and sodium azide [21]. Glutathione reductase (GR) activity was assayed by following the oxidation of NADPH by GSSG [19]. Glutathione-S-transferase (GST) activity was determined from the rate of increase in conjugate formation between reduced glutathione and 1-chloro-2,4-dinitrobenzene [22]. All the measured oxidant/antioxidant parameters were corrected with total protein levels in the hepatic homogenate, and were measured using a spectrophotometer (S1200, Unico, USA).

### Histological and histochemical examinations

Liver sections were fixed in 10% neutral buffered formalin, processed using the paraffin-embedding technique, and then sectioned for staining. Hematoxylin and eosin stain was used for general histological examination [23], Picrosirius red stain for collagen identification [24], and Periodic acid Schiff (PAS) for glycogen content [23]. Examination and photography were carried out utilizing a digital camera (Toup Tek ToupView, Copyrightc 2019, Version:x86, Compatible: Windows XP/Vista/7/8/10, China), ImageJ software, and a computer connected to a light microscope (Olympus CX31, Japan).

### Immunohistochemistry of cleaved caspase-3 and Nrf2

Formalin-fixed liver tissues were put in 10% neutral buffered (pH 7.2). Paraffin-embedded tissues were sectioned, cleared, and rehydrated in a grade of ethanol solutions (100% − 70%) and rinsed in water. Extraction of antigens was done by boiling the slides in 1 mM ethylenediaminetetraacetic acid for 10 min, and emerging sections in 3% H_2_O_2_ for 10 min. Each section was put in a blocking solution at room temperature for one hour. The primary cleaved caspase-3 antibody (1:1000) (Novus Biologicals, LLC, USA) and anti-Nrf2 antibody (1:500) (GeneTex, Inc. North America) were then added for 24 h, followed by the secondary antibodies (1:5000) for two hours. After establishing the reaction with 3,3′-diaminobenzidine for 2–3 min, the sections were stained with hematoxylin for 2–5 min [25].

### Statistical analysis

Data were represented as mean ± standard error of the mean (SEM). The results were analyzed by one-way analysis of variance (ANOVA) followed by Duncan post-test using SPSS program version 16 (SPSS Inc., Chicago, USA). Differences of *p* < 0.05 were considered to be statistically significant.

## Results

### Effects of GA on liver function parameters in UA-intoxicated rats

Exposure to UA resulted in a significant increase in AST, LDH, total protein, globulin, and glucose, with no significant change in ALT and albumin. Supplementation with GA before UA intoxication normalized the AST, LDH, and glucose, although total protein and globulin became higher than the control group. There was no significant change when comparing ALT and albumin in the UA group with the GA + UA group (Table 1). UA-challenged rats exhibited hypercholesterolemia and hypertriglyceridemia compared to the control group. A significant reduction in HDL-C and a significant elevation in LDL-C and VLDL were found in the UA-intoxicated rats. GA intervention failed to significantly improve the studied lipid profile compared to the UA group (Table 2).

**Table 1:** The effect of gallic acid on the plasma liver function parameters following uranyl acetate-induced liver dysfunction in rats

Parameters/Groups	Control	UA	GA + UA	*P* value
Plasma ALT activity (U/L)	9.007 ± 0.389	8.604 ± 0.222	9.063 ± 0.206	0.474
Plasma AST activity (U/L)	0.141 ± 0.011	0.222 ± 0.020^#^	0.146 ± 0.017^&^	0.0150
Plasma LDH activity (U/L)	24.645 ± 3.170	227.880 ± 76.316^#^	65.410 ± 9.306^&^	0.011
Plasma albumin level (g/dl)	2.836 ± 0.106	2.939 ± 0.061	2.954 ± 0.188	0.785
Plasma globulin level (g/dl)	1.790 ± 0.159	3.850 ± 0.524^#^	4.422 ± 0.813^Ω^	0.004
Plasma TP level (g/dl)	4.693 ± 0.205	6.375 ± 0.613^#^	7.536 ± 0.800^Ω^	0.009
Plasma glucose level (mg/dl)	87.594 ± 15.807	193.892 ± 20.750^#^	116.462 ± 22.152^&^	0.003

UA: uranyl acetate; GA: gallic acid; AST: aspartate aminotransferase; ALT: Alanine aminotransferase; LDH: lactate dehydrogenase; TP: Total protein

Results are expressed as mean ± SEM of 6 rats per group (One-way ANOVA followed by Duncan post-test)

#= significant difference between UA and the control groups

&= significant difference between GA + UA and UA groups

Ω = significant difference between GA + UA and the control groups

**Table 2:** Effect of gallic acid on the plasma lipid profile following uranyl acetate-induced liver dysfunction in rats

Parameters/Groups	Control	UA	GA + UA	*P* value
Plasma TC level (mmol/l)	65.812 ± 3.897	92.943 ± 6.280^#^	97.534 ± 5.432^Ω^	0.001
Plasma TG level (mg/dl)	54.456 ± 2.904	85.737 ± 8.115^#^	99.373 ± 6.262^Ω^	0.001
Plasma HDL-C level (mg/dl)	41.167 ± 2.212	24.700 ± 1.327^#^	22.642 ± 1.217^Ω^	0.000
Plasma LDL-C level (mg/dl)	24.453 ± 1.314	36.600 ± 1.621^#^	40.755 ± 2.190^Ω^	0.000
Plasma VLDL level (mg/dl)	13.162 ± 0.779	18.643 ± 1.089^#^	19.475 ± 0.794^Ω^	0.000

UA: uranyl acetate; GA: gallic acid; TC: total cholesterol; TG: triglycerides; HDL-C: high-density lipoprotein cholesterol; LDL-C: low-density lipoprotein cholesterol; VLDL: very-low-density lipoprotein

Results are expressed as mean ± SEM of 6 rats per group (One-way ANOVA followed by Duncan post-test)

#= significant difference between UA and the control groups

Ω = significant difference between GA + UA and the control groups

### Effects of GA on plasma total antioxidant capacity and hepatic redox balance in UA-intoxicated rats

A significant reduction was observed in plasma TAC in the UA group compared to the control group. Liver tissues from UA-exposed rats demonstrated a significant increase in MDA and GSSG and a significant decrease in NO, SOD, GSH, GST, GR, and GPx. GA supplementation effectively returned plasma TAC and hepatic MDA, SOD, GSH, and GST to the control levels. The hepatic GR and GPx in the UA + GA group were significantly improved compared to the UA group, but they were still significantly lower than the UA group. The hepatic NO in the GA + UA group was significantly higher than the control group. The hepatic GSSG was significantly reduced in the GA + UA group but still significantly higher than the control group (Table 3).

**Table 3:** Effect of gallic acid on oxidant/antioxidant parameters following uranyl acetate-induced liver dysfunction in rats

Parameters/Groups	Control	UA	GA + UA	*P* value
Liver MDA level (nmol/mg protein)	3.649 ± 0.454	5.082 ± 0.558^#^	2.820 ± 0.413^&^	0.015
Liver NO level (nmol/mg protein)	96.138 ± 8.952	68.085 ± 6.149^#^	149.783 ± 11.535^&Ω^	0.000
Plasma TAC (nmol/ml)	0.236 ± 0.043	0.069 ± 0.007^#^	0.192 ± 0.032^&^	0.005
Liver SOD activity (nmol/mg protein)	20.483 ± 1.332	7.077 ± 1.691^#^	19.230 ± 1.878^&^	0.001
Liver GSH level (nmol/mg protein)	15.385 ± 0.614	13.010 ± 0.363^#^	15.412 ± 0.587^&^	0.004
Liver GSSG level (nmol/mg protein)	0.372 ± 0.010	0.595 ± 0.017^#^	0.446 ± 0.013^&Ω^	0.000
Liver GST level (µmol CDNB-GSH conjugate formed/min/mg protein)	62.969 ± 2.226	57.244 ± 2.023^#^	40.071 ± 1.416^&^	0.000
Liver GR activity (nmol of GSSG utilized/min/mg protein)	16.289 ± 0.842	8.144 ± 0.421^#^	13.031 ± 0.674^&Ω^	0.000
Liver GPx activity (nmol of GSH oxidized/min/mg protein)	254.577 ± 5.732	178.204 ± 4.012^#^	229.119 ± 5.158^&Ω^	0.000

UA: uranyl acetate; GA: gallic acid; MDA: malondialdehyde; NO: nitric oxide; TAC: total antioxidant capacity; SOD: superoxide dismutase; GSH: reduced glutathione; GSSG: oxidized glutathione; GST: glutathione-S-transferase; CDNB: 1-chloro-2,4-dinitrobenzene; GR: glutathione reductase; GPx: glutathione peroxidase

Results are expressed as the mean ± SEM of 6 rats per group (One-way ANOVA followed by Duncan post-test)

# = significant difference between UA and the control groups

& = significant difference between GA + UA and the UA groups

Ω = significant difference between GA + UA and the control groups

### The effects of GA on the histological features of the liver of UA-intoxicated rats

**Fig. 2:**
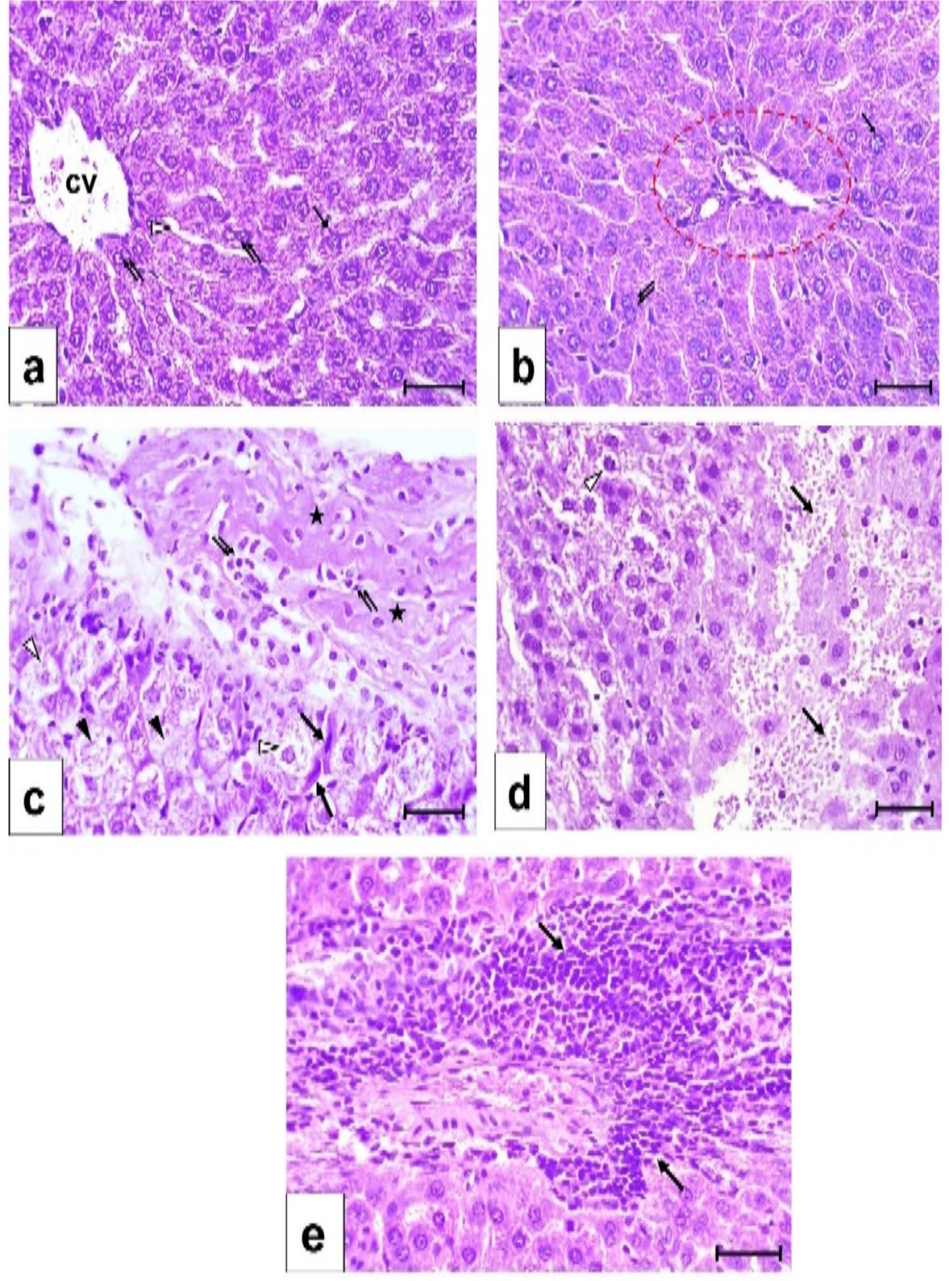
Photomicrographs in the liver sections stained by H&E, bars = 50 μm (**a**) In control group showing a central vein (cv) from which mono- (↑) and binucleated (↑↑) hepatocytes are radiating in cords. The hepatocytes are with rounded vesicular nuclei and granular cytoplasm. Blood sinusoids are between the hepatic cords (Δ). (**b**) In control group showing a portal area (red circle). Mono- (↑) and binucleated (↑↑) hepatocytes are with rounded vesicular nuclei and granular cytoplasm. (**c**) In UA group showing massive fibrotic area (asterisk) enclosing connective tissue cells (↑↑). Hepatocytes are with pale vacuolated cytoplasm and irregular nuclei (Δ). Features of Karyolysis observed in some hepatocytes (▲). Some oval cells with dense flat nuclei noticed between hepatocytes (↑). (**d**) In UA group showing extravasated blood cells in between hepatocytes (↑). Some hepatocytes are with vacuolated cytoplasm and dense nuclei (Δ). (**e**) In UA group showing massive cellular infiltration (↑) around the portal area

**Fig. 3:**
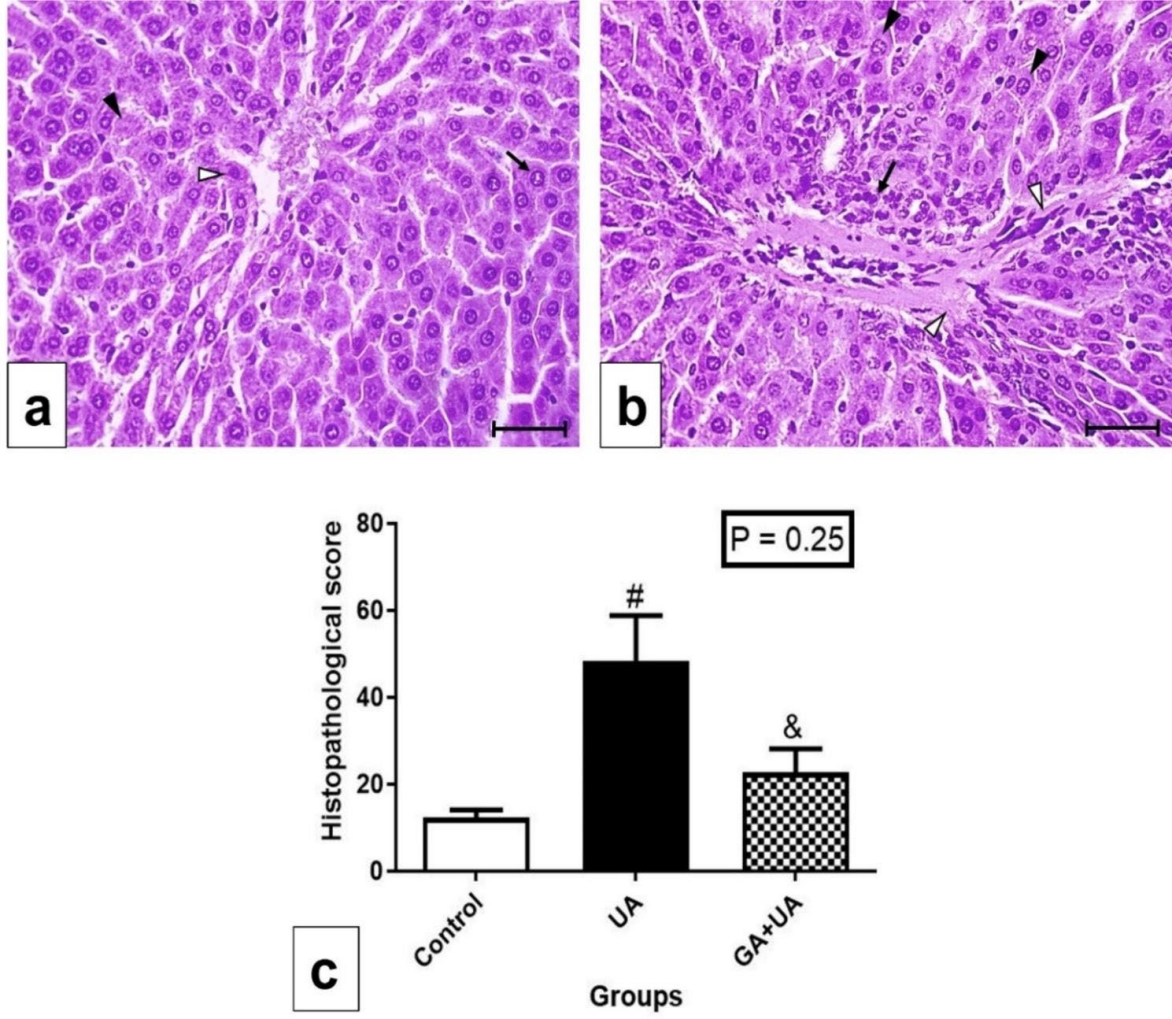
Photomicrographs in the liver sections of GA + UA group stained by H&E, bars = 50 μm (a&b). (**a**) Showing nearly normal appearance of hepatocytes with rounded vesicular nuclei (↑). Few cells are with condensed nuclei (Δ) and the others showing Karyolysis (▲). (**b**) Showing cellular infiltration around the portal area (↑), and appearance of some fibers (Δ). The majority of hepatocytes are nearly normal (▲). (**d**) Liver histopathological score for all the experimental groups. Results are expressed as mean ± SEM of 3 rats per group (One-way ANOVA followed by Duncan post-test). # significant difference between UA and the control groups. & significant difference between GA + UA and UA groups

Hematoxylin and Eosin staining of liver tissue from the control group revealed normal structure (Fig. 2a,b). The hepatocytes radiated in cords from the central vein. Blood sinusoids were located in between these cords. The hepatocytes contained one or two rounded vesicular nuclei and granulated cytoplasm. The portal area included the hepatic artery, portal vein, and bile ductule (Fig. 2b). UA exposure led to degenerated hepatocytes characterized by irregular nuclei and vacuolated cytoplasm. Fibrotic areas with cellular infiltration and extravasated blood cells were observed (Fig. 2c,d,e). GA supplementation prior to UA exposure resulted in nearly normal hepatocytes, with minimal fibrosis and cellular infiltration (Fig. 3a,b). Histopathological scoring confirmed the significant increase in degenerative parameters (vacuolated cytoplasm, irregular nuclei, karyolysis, hemorrhage, and cellular infiltration) in the UA group, while the GA + UA group exhibited insignificant changes compared to the control group (Fig. 3c).

### The effects of GA on the collagen deposition and glycogen content in the liver of UA-intoxicated rats

**Fig. 4:**
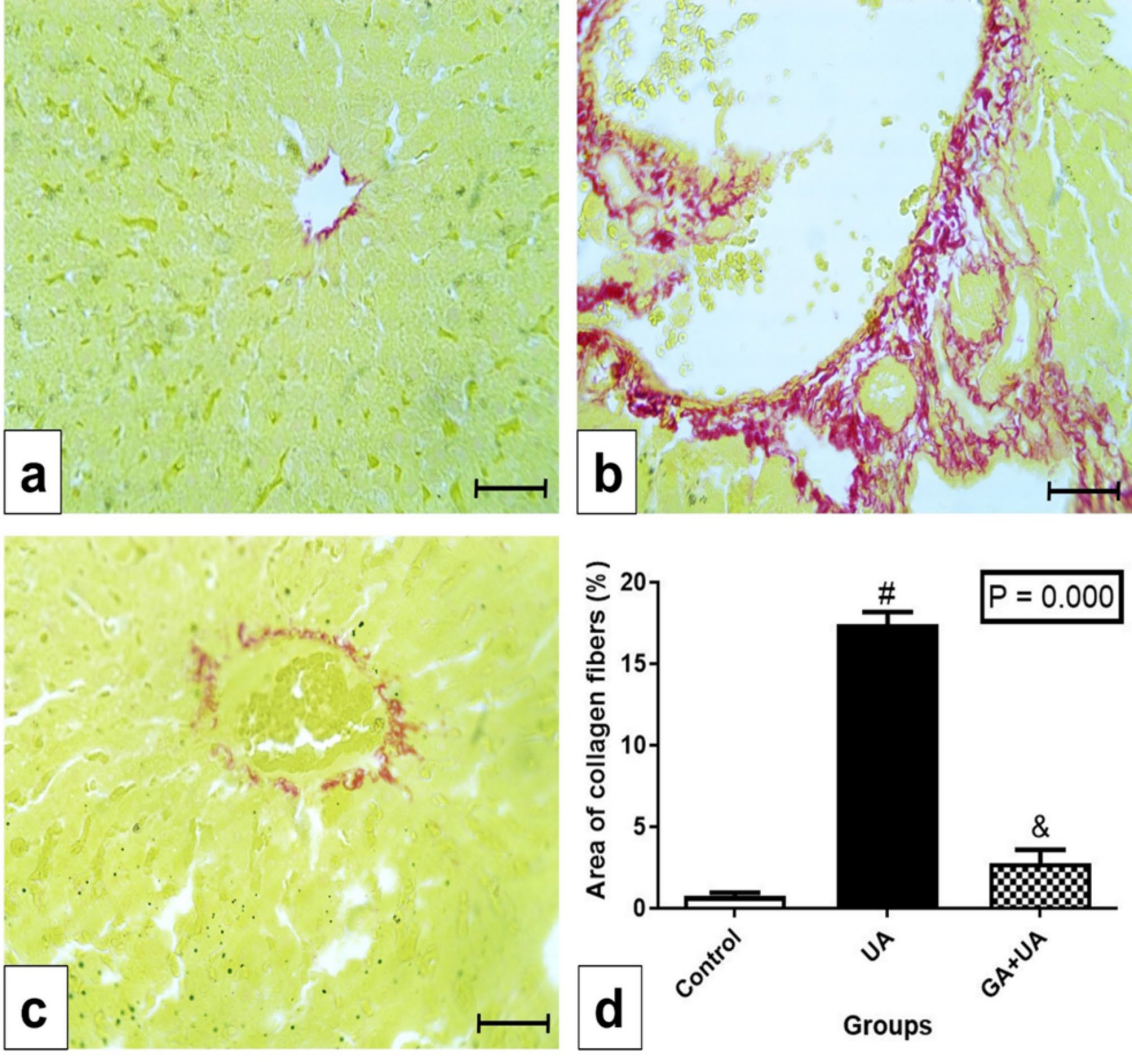
Collagen fibers examination in the experimental groups. (**a**-**c**) photomicrographs of liver sections stained by Picrosirius red stain, bar = 50 μm. (**a**) In control group, showing tiny amount of collagen fibers around the central vein. (**b**) In UA group, showing huge amount of collagen fibers represented by the red color. (**c**) In GA + UA group, showing few amounts of collagen fibers nearly as those of control group. (**d**) Percentage of area of collagen fibers in the different experimental groups. Results are expressed as mean ± SEM of 3 rats per group (One-way ANOVA followed by Duncan post-test). # significant difference between UA and the control groups. & significant difference between GA + UA and UA groups

Examination of collagen fibers by Picrosirius red stain in the control group showed a tiny amount around the central vein (Fig. 4a). Huge amount of collagen fibers was observed in the UA group, evidenced by the red color (Fig. 4b). A significant increase in the percentage of collagen area in the UA group was found compared to the control group (Fig. 4d). GA supplementation mitigated collagen deposition, resembling the control group (Fig. 4c). The percentage of the area of collagen amount in the different experimental groups was represented in Fig. 4d.

**Fig. 5:**
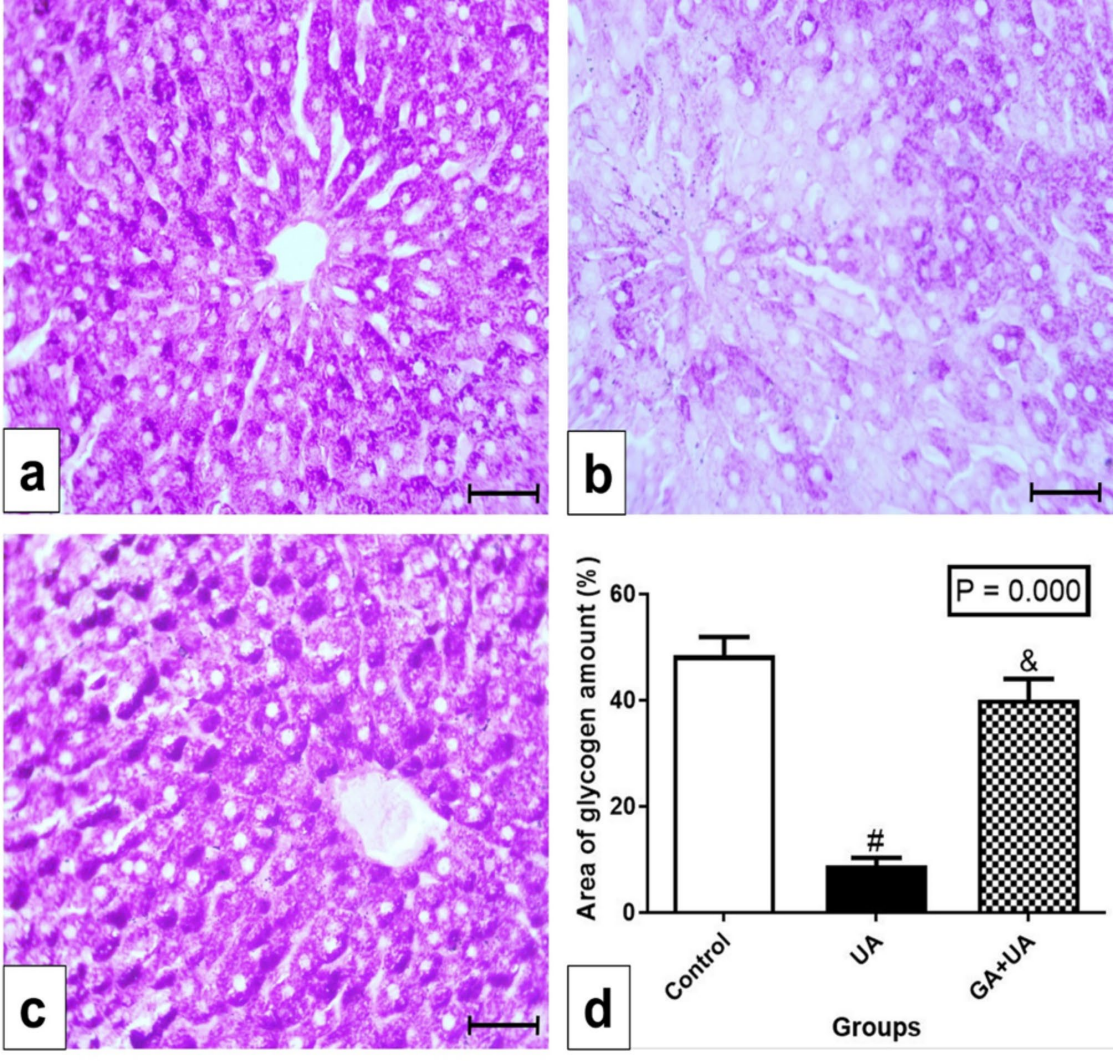
Glycogen examination in the experimental groups. (**a**-**c**) photomicrographs of liver sections stained by Periodic acid–Schiff stain (PAS), bar = 50 μm. (**a**) In control group, showing great amount of glycogen represented as the positive reaction of PAS. (**b**) In UA group, showing noticeable depletion in glycogen content in most of the hepatocytes. (**c**) In GA + UA group, showing positive PAS reaction resembling those of control group. (**d**) Percentage of area of glycogen amount in the different experimental groups. Results are expressed as mean ± SEM of 3 rats per group (One-way ANOVA followed by Duncan post-test). # significant difference between UA and the control groups. & significant difference between GA + UA and UA groups

Glycogen content examination by PAS stain in the control group showed a great amount of glycogen content (Fig. 5a). Noticeable depletion in the glycogen content in most of the hepatocytes was observed in the UA group (Fig. 5b). GA supplementation restored glycogen content to levels similar to the control group (Fig. 5c). The percentage of the area of glycogen amount in the different experimental groups was represented in Fig. 5d.

### The effects of GA on the immunohistochemistry of cleaved caspase-3 in the liver of UA-intoxicated rats

**Fig. 6:**
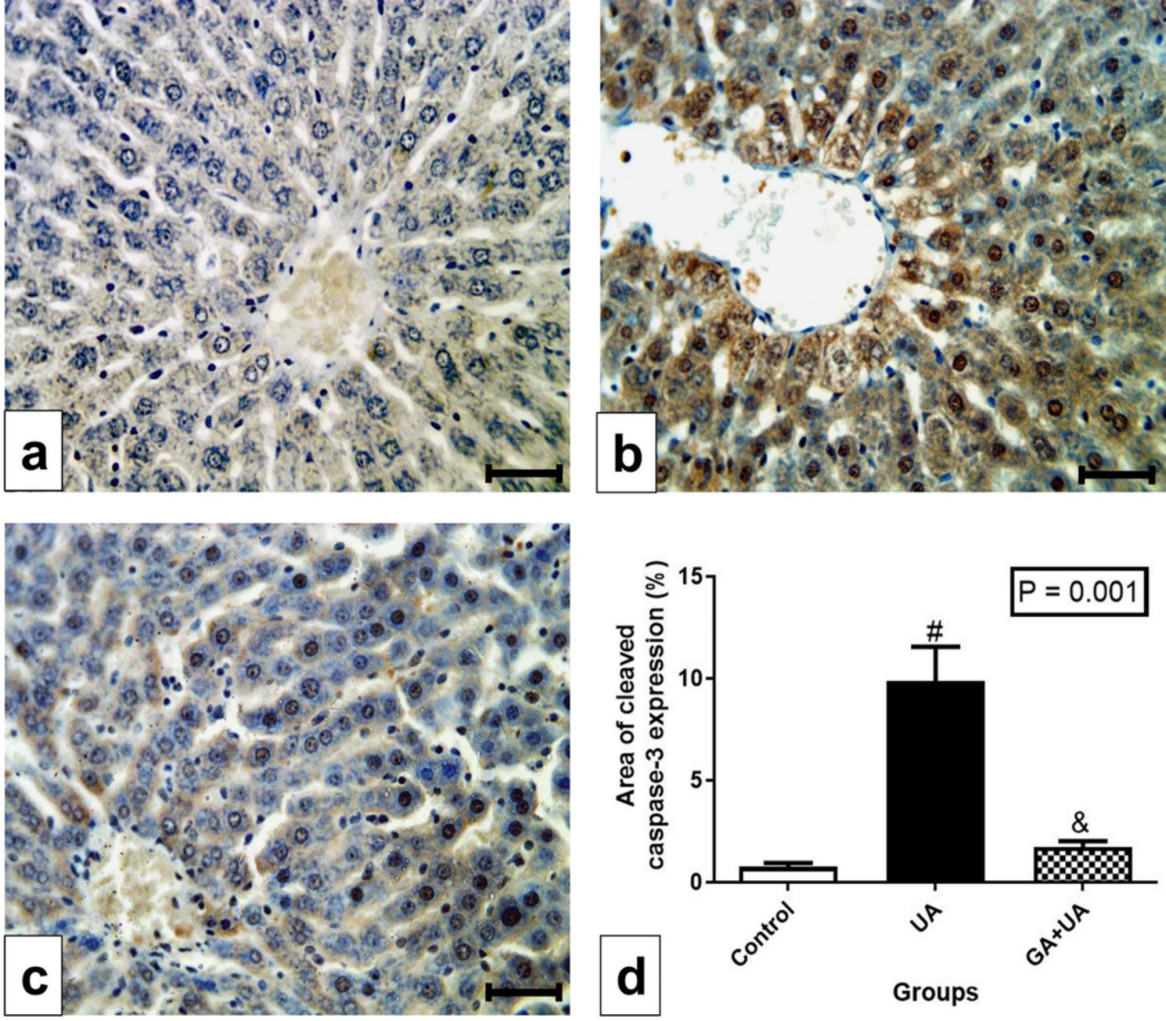
Immunohistochemical detection of cleaved caspase-3 protein in the liver. (**a**-**c**) Photomicrographs of liver sections of rats from the experimental groups, bar = 50 μm. (**a**) In control group, showing negative immunoreaction for cleaved caspase-3. (**b**) In UA group, showing highly positive immunoreaction as represented by brown color specially in the nuclei of hepatocytes. (**c**) In GA + UA group, showing negative immunoreaction in most of the hepatocytes except few ones still with brown nuclei. (**d**) Percentage of area of cleaved caspase-3 protein expression in the different experimental groups. Results are expressed as mean ± SEM of 3 rats per group (One-way ANOVA followed by Duncan post-test). # significant difference between UA and the control groups & significant difference between GA + UA and UA groups

Immunohistochemical analysis of cleaved caspase-3 revealed a negative immunoreaction in the control group (Fig. 6a), contrasting the highly positive immunoreaction observed in the UA group (Fig. 6b). GA supplementation showed mostly negative immunoreaction, with minimal positive staining (Fig. 6c). The area of cleaved caspase-3 protein expression significantly increased in the UA group compared to the control group, while the GA group exhibited insignificant changes compared to the control group (Fig. 6d).

### The effects of GA on the immunohistochemistry of Nrf2 in the liver of UA-intoxicated rats

**Fig. 7:**
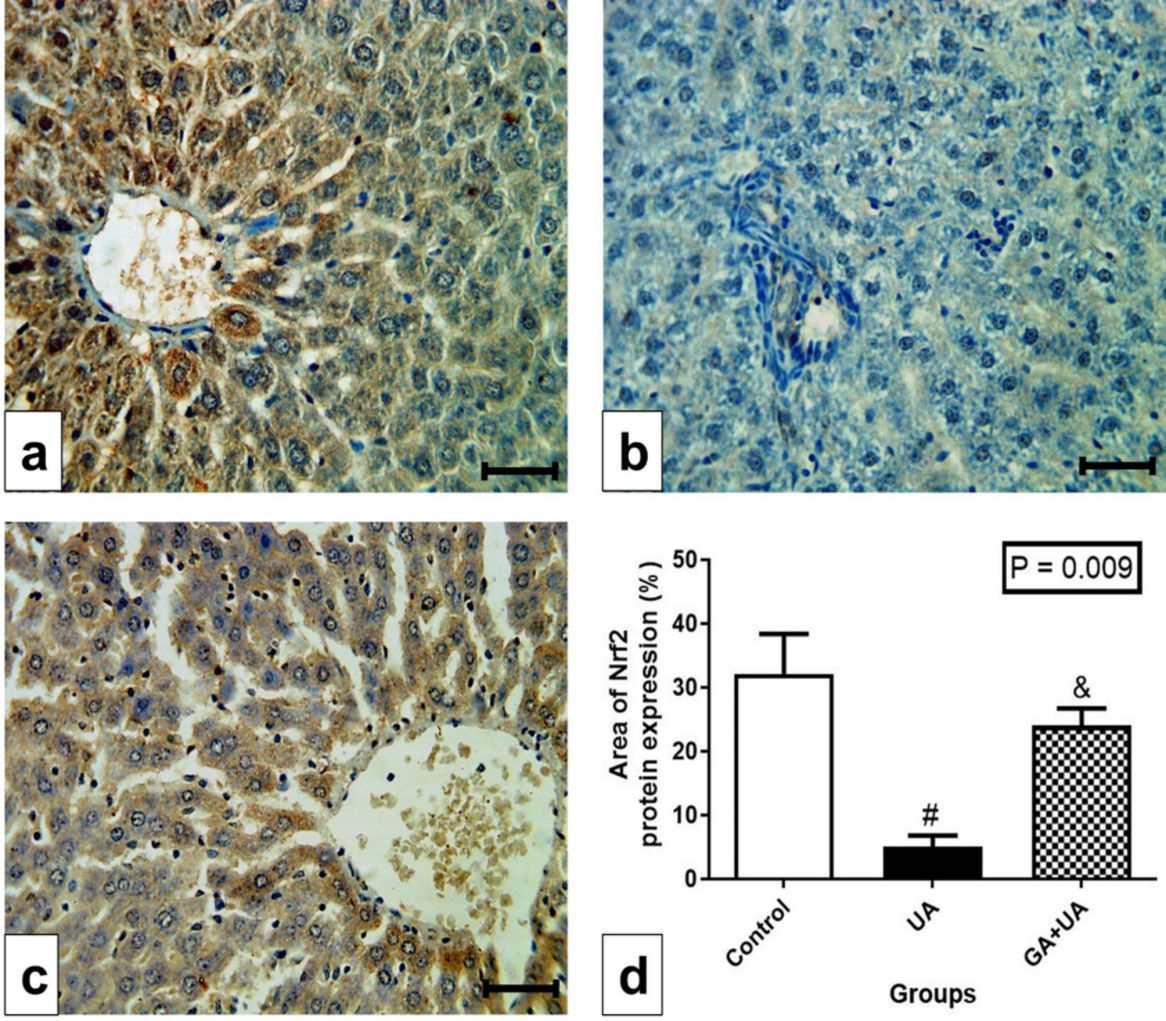
Immunohistochemical detection of Nrf2 protein in the liver. (**a**-**c**) Photomicrographs of liver sections of rats from the experimental groups, bar = 50 μm. (**a**) In control group, showing positive immunoreaction of Nrf2 as represented by the brown color. (**b**) In UA group, showing negative immunoreaction (**c**) In GA + UA group, showing positive immunoreaction. (**d**) Percentage of area of Nrf2 protein expression in the different experimental groups. Results are expressed as mean ± SEM of 3 rats per group (One-way ANOVA followed by Duncan post-test). # significant difference between UA and the control groups. & significant difference between GA + UA and UA groups

Positive immunoreaction of Nrf2 was observed in the control group (Fig. 7a), while UA-exposed rats exhibited negative immunoreaction (Fig. 7b). GA supplementation resulted in positive immunoreaction similar to the control group (Fig. 7c). The area of Nrf2 protein expression significantly decreased in the UA group compared to the control group, with GA supplementation increased it to a level resembling that of the control group (Fig. 7d).

## Discussion

The significant increase in plasma AST and LDH activities mirrors observations from a previous study [4]. In our experimental model, the oxidative burden triggered by UA led to hepatocyte cytolysis, causing the release of cytosolic enzymes into the bloodstream. The elevation in AST activity can be linked to increased production of Krebs cycle intermediates, contributing to fueling gluconeogenesis to meet cellular metabolic demands while maintaining antioxidative capacities to counteract redox imbalances [26]. The increased LDH activity is associated with lactic acidosis, which is implicated in apoptosis through opening the mitochondrial permeability transition pore and inducing cytosolic Ca^2+^ bursts that activate caspases [27]. Conversely, GA effectively normalized plasma AST and LDH activities, consistent with findings in paraquat-induced hepatotoxic rats [28]. The hepatoprotective mechanisms of GA involve hindering the access of oxygen-derived species to the lipid bilayer, reversing oxidative/nitrosative-mediated membrane disruptions, and stabilizing tight junctions and epithelial barriers [29–31].

The UA-associated hyperglobulinemia in our experimental irradiated rats aligns with increased serum immunoglobulin levels observed in orally supplemented mice [32]. This suggests enhanced B cell differentiation to boost specific immunity against xenobiotic contamination or potential impairment in the ability of the liver to clear immunoglobulins from circulation [33, 34]. As part of a compensatory response to reactive damaging molecules, accelerated protein generation supports the biosynthesis of antioxidants and cytoprotective agents [35]. The hyperproteinemia observed in the UA group contradicts findings by Zimmerman and colleagues [36], who reported no significant change in total protein. However, it could serve as a symptomatic marker of hepatic dysfunction [37]. Hyperproteinemia may instigate generation of reactive free radicals and activation of programmed cell death through the endoplasmic reticulum-calcium ion signaling pathway [38] following UA exposure. It was hypothesized that the increase in albumin and ALT by hepatocellular injury was masked by its reduced production due to extensive fibrosis [26, 39], resulting in an insignificant change in ALT and albumin following UA exposure.

Similar to gamma-irradiated rats [40], our experimental irradiated model displayed a marked increase in plasma glucose levels, attributed to mobilization of hepatic glycogen reserves as confirmed histologically. Reduced renal glucose excretion, decreased beta cell number, and impaired glucose uptake [41–43] contribute to this hyperglycemic state. Hyperglycemia-induced overproduction of reactive oxidants may disrupt endothelial tight junctions and the barrier function, leading to leakage of blood from vasculature into surrounding tissues [44]. UA-associated hyperglycemia appears to down-regulate gene expression of Nrf2 and its regulators [45]. Additionally, it down-regulates anti-apoptotic proteins, up-regulates pro-apoptotic factors, and promotes cytochrome c translocation from mitochondria to the cytosol [46]. In contrast, GA supplementation restored glucose homeostasis as demonstrated by Variya and colleagues [47], achieved by delaying intestinal glucose absorption, enhancing beta-cell insulin secretion, and encouraging glucose uptake and peripheral insulin sensitivity [48].

The disturbances in lipid profile observed in the UA group are similar to the findings in gamma-irradiated rats [49]. Elevated activity of hepatic metabolizing enzymes responsible for fatty acid synthesis and mobilization contributes to radiation-induced hyperlipidemia [49]. Up-regulation in the transcript levels of sterol regulatory element-binding protein 1c might participate in this effect [50]. TG enrichment of HDL particles, enhancement of hepatic lipase activity, and inhibition of hepatic production of apolipoprotein A-1 may be responsible for the drop in plasma HDL-C [51]. Damage to the pancreas is a leading cause of inhibiting lipoprotein lipase [52], closely associated with the observed lipoprotein patterns [53]. A two-way relationship exists between hyperlipidemia and hyperglycemia. Hyperlipidemia promotes insulin resistance by blocking insulin signals and destroying pancreatic beta cells, giving rise to hyperglycemia [54]. As a consequence of excess glucose loading, lipid metabolism is impaired. For instance, glucose can be converted to fatty acids and cholesterol through *de novo* lipid biosynthesis pathways, and excessive lipids are secreted in lipoproteins or stored in lipid droplets [55]. Hyperglycemia can predispose to hypercholesterolemia by up-regulating 3-hydroxy-3-methylglutaryl-coenzyme A reductase, and hamper fecal cholesterol excretion and bile acid biomanufacturing [56, 57]. Peroxidation of membrane phospholipids exacerbates cholesterol biogenesis in the liver and other organs through overgeneration of peroxides and disruption of membrane structure-function attributes [58]. Hyperlipidemia induces hepatic oxidative stress, inflammation, and apoptosis [59]. The current GA intervention dosage and duration may be insufficient to counter UA-associated hyperlipidemia, as observed in atherosclerosis-prone apolipoprotein E knockout mice fed a high-fat Western-type diet [60]. Factors affecting gut microbial community and xenobiotic detoxification systems may play a dominant role in modulating the stability, absorption, and metabolism of phytochemicals [61].

The substantial increase in lipid peroxidation end products after UA intoxication concurs with findings by Yuan et al. [4]. UA stimulates excessive free radical production while inhibiting the intracellular redox stabilizing network in rat hepatocytes [62]. This impairment not only affects polyunsaturated fatty acids but can also impact other biological macromolecules, thereby affecting cellular membrane levels and subcellular components [63]. Our work, along with others [64], confirms the ability of GA to counteract the lipid peroxidation cascade in hepatic tissues due to its free radical scavenging properties. GA suppresses the Fenton reaction, which reduces the production of free radicals and the amount of iron available to combine with oxygen to initiate lipid peroxidation [65].

Matched with the depletion of NO in the testicular tissues of UA-exposed rats [8], our finding revealed a remarkable exhaustion of hepatic NO owing to a reduction in NO-secreting cells and inducible NO synthase activators and elevation in NO inhibitors [32]. Reduced NO bioavailability could result from its binding with superoxide radicals to form peroxynitrite or uncoupling of nitric oxide synthase under oxidative stress, further exacerbating the redox imbalance [66]. Disturbances in lipid metabolism in our irradiated model could contribute to NO depletion through mechanisms including L-arginine exhaustion (a key player in NO synthesis), NO synthase dysfunction, increased NO turnover, limited vascular response to its vasodilatory effects, and impaired translocation to target tissues [67]. The elevation in apoptotic signaling and reduction in cell proliferation capacity often correlate with a deficit in NO formation. This is evident from the cytoprotective properties of NO through S-nitrosylation of apoptotic mediators [68]. In contrast, GA supplementation increased hepatic NO levels, surpassing even control levels. This effect is due to the ability of GA to slow NO turnover and enhance endothelial NO synthase phosphorylation [69, 70]. Increased NO levels activate the pentose-phosphate pathway [71], a major NADPH producer that regenerates reduced GSH from its oxidized form. The elevation in NO levels correlates with the increase in SOD activity, suggesting a causal link. As SOD catalyzes the dismutation of superoxide radicals into molecular oxygen and hydrogen peroxide, heightened SOD activity clears superoxide anions, thus preserving NO bioavailability [72]. Additionally, NO is essential for up-regulating SOD expression, preventing superoxide radical-mediated NO degradation [73].

Similar to the findings of Hao et al. [74] and Pourahmad et al. [62], GSH redox network was altered in the UA group. This outcome indicates a failure in a critical component of the xenobiotic detoxification system [75], rendering the hepatic microenvironment more susceptible to the radiological hazards of UA. Lactic acidosis prompts metabolic reprogramming to enhance NADPH synthesis, shifting the glutathione redox couple towards the oxidized form to counter reactive oxidative stress [76]. Moreover, utilization of glutamine for ATP production under acidic stress contributes to the depletion of other glutamine-related metabolites, including GSH [76]. Reactive oxidant generation caused by UA triggers GSH oxidation and inactivation of GSH-related enzymes [77]. GST eliminates lipid peroxidation end-products and contaminants-derived electrophilic compounds [78, 79], thereby preventing cell membrane damage. The reduction in GST could be due to the down-regulation of its gene expression [80]. GSH is necessary for ensuring the continuation of thiol group reduction in mitochondrial membrane proteins [81]. When these thiol groups are oxidized, the pore complex undergoes structural modifications, resulting in a mitochondrial permeability transition that is a leading factor in both necrosis and apoptosis mechanisms [82]. Restoration of hepatic GSH redox cycle in the GA + UA group is compatible with what happened in doxorubicin-induced hepatotoxic [77] and streptozotocin-induced diabetic rats [65]. The increase in hepatic GSH levels in UA-irradiated rats pre-supplemented with GA is attributed to the up-regulation of gamma-glutamylcysteine synthetase, a rate-limiting enzyme in GSH biosynthesis [83]. Activation of Nrf2 results in increased transcript abundance of downstream antioxidants-related genes, including those belonging to the GSH redox system [84].

Total antioxidant capacity (TAC) provides a holistic view, accounting not only for the sum of individual antioxidants but also for their complex interactions [85]. The normalization of TAC reflects the ability of GA to restore the overall body’s redox balance. The improved redox potency of hepatic tissue in the GA + UA group is attributed to increased transcript levels of antioxidants and scavenging of free radicals [86, 87]. This is supported by the increase in Nrf2 immuno-expression, a crucial transcription factor that plays a pivotal role in defending against peroxidative damage by up-regulating various enzymatic antioxidants. The Nrf2 signaling pathway is a critical mediator in controlling the transcription of numerous antioxidant genes, including enzymes involved in GSH and SOD synthesis [88]. GA disrupts the interaction between kelch-like ECH-associated protein 1 and Nrf2 in drug-induced hepatic dysfunction, leading to increased nuclear translocation of Nrf2 [83]. Sirtuin 1 overexpression resulting from GA supplementation facilitates Nrf2 nuclear translocation, stabilizes Nrf2 protein expression, and enhances nuclear accumulation, DNA binding activity and transcriptional function of Nrf2 [89, 90]. Targeting Nrf2 may offer a promising therapeutic strategy to enhance cellular stability against redox imbalances, a key factor in driving and exacerbating radiation-associated hepatic damage.

The hepatic histoarchitectural deteriorations induced by UA exposure are consistent with other reports [4, 91]. These cellular changes could arise from mitochondrial dysfunction and disruption of oxidative phosphorylation [1]. Vacuolated cytoplasm in the liver could result from dysregulated fatty acid metabolism, leading to neutral fat accumulation, which gets dissolved during tissue preparation, leaving empty unstained vacuoles [92]. Karyolysis in hepatocytes, similar to uranium-contaminated mice [93], is attributed to endonuclease activity secreted by Kupffer cells, causing destructive fragmentation of genomic material [94]. The heightened release of reactive oxygen species, as indicated by increased MDA levels in the UA group, drives the excessive formation of extracellular matrix proteins ensuring optimum conditions for hepatic fibrosis [95]. Hyperlactemia resulting from increased LDH activity triggers transforming growth factor-beta, leading to fibroblast differentiation [96]. The marked occurrence of apoptotic hepatocytes following UA contamination aligns with findings in testicular germ cells [8]. UA triggers genotoxic damage indirectly through single-strand breaks, facilitated by oxidative DNA damage *via* Fenton redox reactions, and directly through covalent binding to DNA [97]. GA excretes antifibrotic activity by reducing hepatic pro-fibrogenic cytokines and blocking hepatic stellate cells activation and proliferation [98]. The anti-apoptotic effect of GA against UA-induced hepatotoxicity corresponds to its protection against ultraviolet radiation-induced damage in zebrafish and human keratinocytes [99]. Scavenging free radicals, reducing transcript levels of Bax and caspase-3, increasing Bcl-2 transcript levels, and enhancing genomic repair [9, 100] underlie the cytoprotective properties of GA.

## Conclusion

Pre-treatment of UA-exposed rats with GA efficiently restored the liver’s redox stability and cyto-functionality by inhibiting lipid peroxidation, up-regulating Nrf2, and suppressing the apoptotic cascade. This discovery holds significant value in guiding the scientific community toward recognizing the beneficial role of natural phytochemicals in mitigating the health risks associated with DU exposure. Further studies are highly recommended to highlight the molecular mechanisms underlying the protective effects of GA against UA intoxication.

## Data Availability

The datasets used and/or analyzed during the current study available from the corresponding author on reasonable request.
